# A Multi-Institutional Informed Consent Proposal as a Prevention Tool for Combined Oral Contraceptive Intake and Thrombotic Risk

**DOI:** 10.3390/jpm13040584

**Published:** 2023-03-27

**Authors:** Marina Vinciguerra, Eliano Cascardi, Bruno Lamanna, Maricla Marrone, Fortunato Pititto, Enrica Macorano, Romualdo Sciorio, Giorgio Maria Baldini, Antonio Malvasi, Andrea Ballini, Gerardo Cazzato, Antonella Vimercati, Senthil Kumaran, Ettore Cicinelli, Salvatore Scacco, Miriam Dellino

**Affiliations:** 1 Unit of Obstetrics and Gynaecology, Department of Biomedical Sciences and Human Oncology, 70132 Bari, Italy; marinamia91@gmail.com (M.V.); miriamdellino@hotmail.it (M.D.); 2 Department of Obstetrics and Gynaecology, “Santa Caterina Novella Hospital”, 73013 Galatina, Italy; 3 Department of Medical Sciences, University of Turin, 10124 Turin, Italy; eliano.cascardi@ircc.it; 4 Pathology Unit, FPO-IRCCS Candiolo Cancer Institute, Str. Provinciale 142 km 3.95, 10060 Candiolo, Italy; 5 Fetal Medicine Research Institute, King’s College Hospital, London SE5 9RS, UK; 6 Section of Legal Medicine, Interdisciplinary Department of Medicine, University of Bari “Aldo Moro”, 70121 Bari, Italy; 7 EFREC (Edinburgh Assisted Conception Programme), “Royal Infirmary of Edinburgh”, Edinburgh EH16 4SA, UK; 8 Momò Fertilife Clinic, 76011 Bisceglie, Italy; 9 Department of Precision Medicine, University of Campania “Luigi Vanvitelli”, 80138 Naples, Italy; 10 Department of Precision and Regenerative Medicine and Jonic Area, University of Bari “Aldo Moro”, 70121 Bari, Italy; 11 Department of Forensic Medicine and Toxicology, All India Institute of Medical Sciences, Mangalagiri 522503, India; 12 Department of Basic Medical Sciences and Neurosciences, University of Bari “Aldo Moro”, 70121 Bari, Italy

**Keywords:** oral contraceptive, contraceptive informed consent, thrombosis risk, venous thromboembolism

## Abstract

Combined oral contraceptives (COC), are among the most widely used contraceptive methods in the world today. Despite the different changes in terms of estrogen/progestogen combinations and dosages, the thromboembolic risk for a woman who takes combined oral contraceptives persists to date. Methods: The review of relevant literature and international guidelines on prescription of combined oral contraceptives made it possible to create a proposal for informed consent to be used for prescribing. Results: The several sections of our consent proposal were designed according to a rationale in order to cover all the aspects presented by worldwide guidelines: how to take, adverse effects, advertisements, extra-contraceptive benefits and effects, a checklist for condition at risk of thromboembolism, the signature of the woman. Conclusions: An informed consent to standardize combined oral contraceptives prescription can improve women’s eligibility, mitigate thromboembolic risk, and assure legal protection to healthcare providers. In this systematic review in particular, we refer to the Italian medical–legal scenario, to which our group of researchers belongs. However, the model proposed was designed in the respect of main healthcare organization guidelines, and it could be easily used by any center in the world.

## 1. Introduction

The use of combined oral contraceptives, also known as COC or simply as a “pill” in common languages, is today one of the most discussed and constantly evolving issues. In 1981, Mclure et al. first stated that COC has the lowest Pearl Index (PI), corresponding to 0.1, in comparison with the other existing contraceptive methods—such as, for example, progesterone-only minipills (PI 1–2), intrauterine devices (PI 1–3), condoms (PI 0.4–1.6), lactation for 12 months (PI 25), and coitus interruptus (PI 9) [1]. Even today, the COC is the most effective method of contraception, comparable only to the tubal ligature (PI 0.04) and vasectomy (PI 0.1), which are however a surgical procedure and irreversible [1,2].This is why, to date, COC is one of the most widely used contraceptive methods in the world, mostly in developed countries, such as the United States, Australia, and the European Union [3,4,5]. In Italy, the most common is the condom (prevalence 39.1%), but the main contraceptive method still remains the pill (prevalence 25.9%) as the most adopted method in all groups of women considered, in pairs or not. Also noteworthy is the constant greater use of pills and condoms among women who are not in couples (34.9% and 33.4%, respectively), compared to those who are (21.3% and 16.1%, respectively), possibly according to the different maternity projects in the two groups [6].

This reflects the additional health effects of COC, which allows them to pre-prescribe therapy along with simple contraception. The main extra-contraceptive benefits of hormone combination contraception are divided into three categories. The first acts on symptoms of the menstrual cycle (dysmenorrhea, heavy menstrual bleeding, premenstrual syndrome, menstrual headache, hyper-androgenic symptoms); the second improves gynecological pathologies, such as adenonomiosis or endometriosis and related consequences [7]; the third prevents effects of pathological conditions (pelvic inflammatory disease, also known as PID, extrauterine pregnancy, ovarian/endometrial/colorectal and other cancers) [8,9].

Hormonal contraception contains a progestin with or without an estrogen, which—from a pharmacological point of view—is a steroid or lipid hormone [3]. Progesterone is the only naturally occurring progestin; most contraceptive progestins, such as levonorgestrel and norethindrone, are synthesized from testosterone [3]. Estrogens enhance contraceptive effectiveness by suppressing gonadotropins and follicle-stimulating hormone, preventing the development of a dominant follicle [5]. However, the most important contribution of estrogens to progestin-based contraceptives is the reduction of irregular bleeding [8]. The estrogen component in most combined hormonal contraceptives is ethinylestradiol [5].

Despite the many benefits, some physicians and even more women still have doubts about the possible side effects associated with taking COC [10]. Actually, the most feared is the risk of thrombosis, which still exists, as claimed by many health organizations around the world [11]. COC-related thrombotic diathesis occurs primarily as venous thromboembolism (VTE), a nosologically entity consisting of deep venous thrombosis, commonly in the thigh or lower leg, or pulmonary embolism. While VTE is a rare event in young women of reproductive age (incidence 1–5/10,000 woman-years), COC increase the overall risk of VTE, increasing its incidence approximately up to 10–15/10,000 woman-years [12,13,14,15,16].

**Figure 1:**
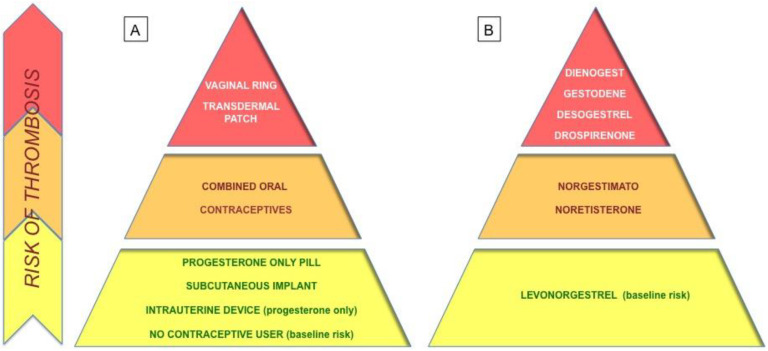
Contraceptive methods and thrombosis risk: The pyramid on the left (**A**) groups the different contraceptive systems, including physiological conditions such as pregnancy, classifying them according to the corresponding risk of thrombotic events (highlighted by the change in color, from yellow to red); the pyramid on the right (**B**) shows the various progestin components of the combined oral contraceptives available on the market classified according to their risk of thrombotic events.

However, VTE risk due to COC intake is still very low and is much lower than the risk during physiological conditions such as pregnancy (approximately 5–20/10,000 woman-years) and the postpartum period (40–65/10,000 woman-years) [17]. COC-related VTE risk is also lower than the corresponding VTE risk with other contraceptive methods (Figure 1A), which also promote an increase of blood clots, such as a transdermal patch or vaginal ring [18,19].

In order to mitigate this risk, it is essential that the prescription of COC consists of comprehensive counseling with the woman, focusing on two main elements. First, the women should know signs and symptoms of VTE, such as swollen or painful calf muscles or shortness of breath, being able to early recognize them and to consult a healthcare provider as soon as possible to improve prognosis. Second, the medical specialist should be sure to detect the current risk factors of any possible individual woman that might contraindicate hormonal contraceptive prescription [12,16]. In this paper, our research group suggests the use of informed consent for prescribing COC to mitigate the risk of VTE, making COC absorption safer.

## 2. Materials and Methods

A thorough search of relevant scientific literature has been carried out on multidisciplinary databases (PubMed, Scopus, Research Gate, Google Scholar) and articles that were published up to 2021 with corresponding updates. The research keys used were combined oral contraceptive, pill, hormone therapy, estrogen, progestogen, contraception, thrombotic risk, and deep venous thrombosis. The literature focused on cases, and cases where incorporation of COC was not related to thrombotic occurrence were overlooked. The search for sources was directed towards all guidelines that investigated the issue. Moreover several studies have been found by sifting through the references of the selected papers and an additional search was conducted on institutional websites for reports, guidelines, opinions, and recommendations released by international and national institutions, such as the World Health Organization, Centers for Disease Control and Prevention, Faculty of Sexual and Reproductive Healthcare of the Royal College of Obstetricians and Gynaecologists, Royal College of Obstetricians and Gynecologists, American College of Obstetricians and Gynecologists, National Institute for Health and Care Excellence, Italian Society of Gynecology and Obstetrics, Italian National Institute of Health.

The search was limited to the English and Italian languages, and all articles have been independently reviewed by three authors (E.C., A.B., and M.D.) to ensure their relevance to this paper; only those articles deemed relevant by at least two of them and with the agreement of the other authors have been ultimately selected.

## 3. Results

**Figure 2:**
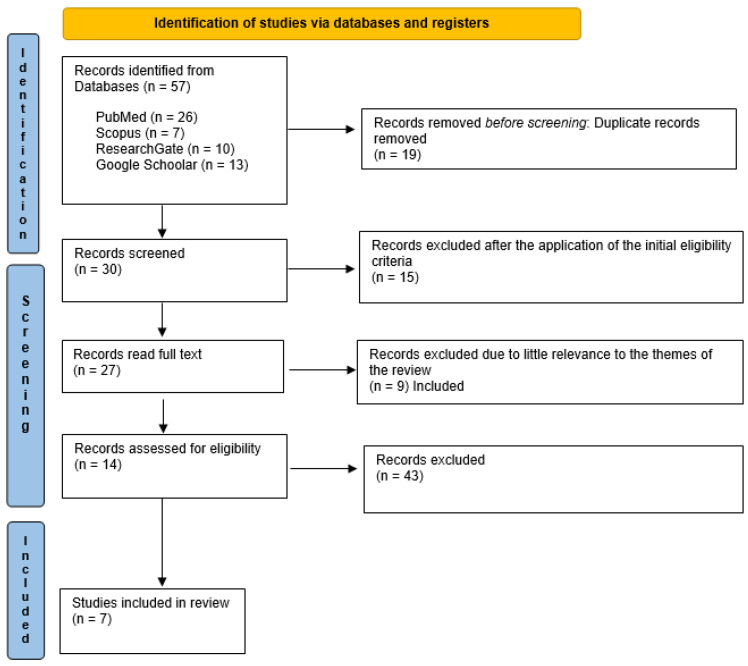
PRISMA flowchart diagram of the inclusion process.

Over the past 12 years, 14 of the 57 papers relating to the problematic subject matter of our study (COC and thrombotic risk) have been selected by the main scientific search engines since they are considered the most comprehensive and up to date. The search in the PubMed, Scopus, Research Gate, and Google Scholar databases resulted in 57 bibliographic sources. The articles that were potentially eligible were 14, of which only 7 fully complied with the present study’s aim criteria, and the related extracted data were included. The entire procedure of identification, selection, and inclusion of the studies has been indicated in the flow chart of Figure 2.

In evaluating the current literature, we have identified seven manuscripts that refer to guidelines, practice guidelines, or briefing notes from global health organizations. These guidelines were used as a basis for realizing our informed consent [3,12,16,17,20,21,22]. These international guidelines agree that assessing medical eligibility for the prescription of COC should not automatically include blood tests, including testing for thrombophilia/hyperlipidaemia/diabetes mellitus/liver function or pelvic/breast examination and cervical screening. However, the force of the recommendation is, instead, mandatory for evaluating the following aspects: medical conditions, lifestyle history, drug history (any prescribed or non-prescribed drug that could affect COC/itself be affected by COC), a recent blood pressure recording (strength of recommendation C), and body mass index (BMI) evaluation (D) [12,20,21,22]. Our informed consent (Supplementary Materials) could be a useful tool which can better define and standardize the prescription of COC and, thus, the corresponding woman’s counsel.

Comprehensive contraceptive counselling should focus on three main objectives: giving detailed and comprehensive information about the method of contraception chosen; assisting and sustaining a woman’s decision-making process professionally [5,23,24]; avoiding a managerial approach in favor of an individualized approach that is respectful and attentive to the preferences, needs, and individual values of women.

Our informed consent, which has been designed in accordance with global guidelines, could ensure good clinical anamnestic assessment as well as good patient information, attaining primary and secondary prevention of VTE risks. Its application could easily stratify the target of low-thromboembolic women and the proper choice of COC prescription, making an objective proof of it without the need of performing any blood exams.

The main section, which consists of our informed consent, is on how to take COC, specific warnings to know before supposing, possible adverse effects, signs and symptoms that appear during COC intake require a timely clinical evaluation, extra-contraceptive health benefits and effects, woman’s data including a checklist of possible VTE risk conditions, and the woman’s signature.

### The Informed Consent for Combined Oral Contraceptive: Our Proposal

The possible informed consent, will address the following points, as reported in Supplementary Materials:

(I) How to take It: The use of COC from the first day of the menstrual cycle, guarantees an intermediate efficacy. If one wants to start taking COC at any time of the cycle, it is recommended to use an additional contraception for 7 days and only with pills containing estradiol valerate (EV) +dienogest (DNG) in the formulation 26 + 2, for 9 days.
(II) Warnings: The first week of contraceptive use, either combined estrogen-progestin oral contraceptive pills (OCPs) or progestogen-only pills (POPs), inhibits ovulation. Delays or forgetting to take pills greatly increase the chances of pregnancy in the first week of the package. The allowed admission time is 12 h; after 24 h, it is advisable to take two tablets together. Risk of inefficiency in case of vomiting within 3 h or excessive diarrhea after 4–6 h of consumption. First clinical examination within 12 months of starting treatment, blood pressure control within six months of starting treatment. COC use should be discontinued 4 weeks before major surgery.
(III) Side effects: The COC is associated with an increased risk of VTE, but it should be considered that in most women, the benefits associated with using COC far outweigh the risk of serious side effects. There is the possibility of water retention, which may cause a weight increase of no more than 2 kg completely reversible upon the suspension of the method. However, the available literature does not identify the effect of COC on weight. Sensations of heavy legs, headache during taking or pause, mastodynia, nausea, rare vomiting, or changes in sexual desire may occur. Most side effects are reduced from the second cycle, often resolving spontaneously within 5 months. Occasional bleeding may occur. Have a specialized medical assessment for side effects from any entity. Please note: COC causes an increased risk of venous and arterial thrombosis ranging from 5 to 12 per 10,000 women each year depending on the type of COC taken. Therefore, it is recommended to alert the doctor and to go to the emergency department if there are signs or symptoms attributable to this event (swollen arms or ankles, legs tingling, lower extremity edema, erythromelalgia, dyspnea, etc.). In this regard, it is recommended to periodically reassess individual risk factors, such as age, tobacco use, obesity, etc.
(IV) Symptoms Or Diagnoses Requiring Clinical Assessment During COC.
  (a) Urgent action: chest pain, shortness of breath, hemoptysis, pain in one leg (usually the calf or inner thigh), swelling in the leg or arm, numbness or weakness on one side of the body, sudden change in your mental state.
  (b) Outpatient care: breast node, secretion from the nipple, nipple inversion, changes in the breast skin; new onset migraine, new sensory or motor symptoms in the hour preceding the onset of migraine; persistent atypical vaginal bleeding; arterial hypertension, increase in BMI >35 kg/m2, migraine or migraine with aura, deep venous thrombosis or pulmonary embolism, blood clotting abnormality, antiphospholipid antibodies positivity; angina, heart attack, stroke or peripheral vascular disease, atrial fibrillation, cardiomyopathy; mutation of the gene BRCA1-2 or breast cancer, liver tumor, gallbladder calculus.
(V) Extracontraceptive Benefits: Hormonal contraception has a protective effect on genital integrity and fertility, resulting in an overall benefit in terms of reproductive health. Reduction of dysmenorrhea and heavy menstrual bleeding, menstrual headaches, hyperandrogenic symptoms, endometriosis, ectopic pregnancy, prevention of ovarian, and endometrial and colon cancers.

## 4. Discussion

In order to suggest our informed consent as a prevention tool for VTE during COC admission, we focused on several points. First, we have developed a checklist with all possible contraindications, outlined in the main international guidelines mentioned above. They are 21 anamnestic criteria, which represent an absolute contraindication to COC intake because they all are attributable to almost one of the following conditions, which alone or together, plus the pill, show an additive and synergize pro-thrombotic effect according to well-known pathophysiologic mechanisms: hepatic dysfunction, cancer, pro-logistic conditions, pregnancy, breastfeeding, and vasculopathies of any types. The checklist allows the physician to ensure they have collected all the necessary anamnestic data at the time of prescription [2]. Secondly, in our informed consent, we decided not only to add several recommendations on how to correctly take the pill, but also a well-detailed description of any possible signs or symptoms needing clinical evaluation. Specifically, in the consent, it is distinguished between urgent conditions, which must be evaluated in the emergency room and conditions whose evaluation can be deferred and must be performed in an outpatient setting. This allows the woman to be more aware of her therapy, stratifying possible side effects by relevance, avoiding unnecessary and excessive worries for the woman and, at the same time, improper hospital access [22].

Finally, the reference to extra-conceptive effects of COC is relevant for two main reasons. On one hand, the woman who chooses COC as a method of contraception is reassured and encouraged on this type of medicine, improving its compliance and, consequently, the effectiveness of the COC. In fact, in the past, the COC hypothesis was interrupted due to misinformation about COC and the link to oncological risk, which was then discredited by the more recent literature [25,26]. On the other hand, the physician should specify on the consent if the COC prescription is not only for a contraceptive aim but if is a therapy for a gynecological condition because this affects the type of COC chosen and the associated VTE risk. In fact, each type of COC has its own VTE risk based on its qualitative and quantitative estrogen–progestogenic composition, as also addressed from the seven included studies, according to the PRISMA flow chart [3,12,16,17,20,21,22].

In particular, education of females, couples, and medical and paramedical staff is one of the priority targets to improve contraception throughout the world [3]. While VTE is rare in young women of reproductive age, combined oral contraceptives increase the risk of VTE. In patients in whom combined hormonal contraception is appropriate, it is reasonable to use any currently available preparation [12]. Recently, the European Medicines Agency’s Committee for Medicinal Products for Human Use (CHMP) has concluded that the benefits of COC in preventing unwanted pregnancies continue to outweigh their risks and that the well-known risk of VTE with all COC is small [16]. Additionally, a previous FDA review concluded that drospirenone-containing birth control pills may be associated with a higher risk for blood clots than other progestin-containing pills [17]. According to guidelines, COC containing ≤30 μg EE (ethinyl-estradiol) in combination with levonorgestrel (LNG) is a reasonable first-line choice of the COC as mere contraception in order to minimize cardiovascular risk [12,16,20,21,22]. In fact, the risk of VTE is linked to the estrogen dose, but the metabolism of EE varies greatly both between individuals and within a single individual. The EE bioavailability of approximately 38% to 48% must be measured at the minimum effective dose [27]. Finally, the estrogenicity of a COC is the amount of both the estrogen and the progestogen contribution and excessive estrogenicity was reported to increase the risk of VTE. The biomarker that best reflects the estrogenicity is the sex-hormone-binding globulin (SHBG), a carrier protein for estrogen and testosterone produced by the liver and whose synthesis is highly estrogen-sensitive [11]. LNG, instead, belongs to the second generation of progestins and is the one with the lowest VTE risk in association with EE in comparison with the other combinations with the same estrogen component (Figure 1B) [22,28]. However, when the COC is a treatment, despite a higher relative risk of VTE, the doctor is allowed to choose a different combination of COC. In the example, dienogest is the progestogenic component of choice for endometriosis despite its relative risk with EA being 1.6 times that of LNG. Instead, drospirenone, like other fourth-generation progestogens, could be selected for patients with clinical and/or biochemical hyperandrogenics, varying its relative risk with EE around 1.5–2.0 more than LNG [22].

In a Cochrane Review, Roach et al. [29] aimed to assess the risk of arterial thrombosis in different types of oral contraceptive pills. The results of this meta-analysis showed that the risk of myocardial infarction or ischemic stroke was 1.6-fold increased in women using COC. The risk was highest for pills containing ≥50 µg of estrogen. The risk of myocardial infarction or ischemic stroke did not differ clearly between progestogen generation in combination with estrogens. However, COCs are especially known to increase the risk of venous thrombosis, and this risk should be kept in mind when counselling women about their choice of contraception [29,30].

When prescribing contraceptives to women, physicians have a central role in screening COC for contraindications, explaining the risks and potential side effects, weighing the risks versus benefits, as well as providing advice on the appropriate use of contraceptives [31]. The physician who prescribes the COC or the pharmacist who dispenses them could provide all women with a formal written risk assessment for contraindications [31]. In addition, we should not ignore the role of self-care; women can self-screen using a checklist of contraindications [32].

Studies show that adults can self-screen for contraindications, but little is known about adolescents. In a recent study [33], the author’s findings suggest that both primary and subspecialty clinicians taking care of patients with a medical contraindication to hormonal contraception, especially estrogen, should take time to specifically educate and counsel their patients regarding their contraindications and impact on birth control options as access to contraception expands. Additionally, this can serve as an opportunity to discuss both pregnancy and contraception through the lens of an adolescents’ and young adults’ (AYAs) medical condition [33], taking also into account the issue to inform AYAs that COC does not protect against sexually transmitted infections (STIs), including *Candida albicans*, HPV, and HIV [34,35,36,37,38,39,40]. It is very important to notice that adolescents consider gynecologists, pediatricians, and other health care providers a highly trusted source of sexual health information.

Women’s voluntary use of contraception is crucial for safeguarding their reproductive rights. Each woman has the right to evidence-based, comprehensive contraceptive information, education, and counselling to ensure informed choice. Women’s contraceptive choices are made in a particular time, societal, and cultural context; choices are complex, multifactorial, and subject to change [20]. Decision-making for contraceptive methods usually requires the need to make trade-offs between the different methods, with advantages and disadvantages of specific contraceptive methods varying according to individual circumstances, perceptions, and interpretations [20].

Further research will identify the risk of VTE associated with another combination of COC—for example, with estradiol-2-valerato—to minimize the relative risk in COC regimens and therapeutic combinations. The idea and design of our informed consent arose not only from the literature and epidemiological relevance but also from the Italian forensic scenario, to which our group of researchers belongs. At the same time, however, we have created a valuable tool across the globe. The Italian legislation specifies that the doctor must provide the patient with clear and complete information on the risks and benefits of therapy. Most of the drugs used for contraception are not strictly therapeutic. Therefore, the risk/benefit ratio should be carefully calibrated according to the patient’s needs and personal desire. The advantage is to protect the conscious decision to have a pregnancy. In this sense, it may generally be understood as the benefit of preventing health damage (psychological, physical, social) resulting from unwanted pregnancy. Those risks have already been described here. In general, the prescribing physician needs to be aware of scientific advances, individual benefits, and risk factors, taking into account the difficulties of medical examination linked to the SARS-CoV-2 pandemic status of the last years [40,41,42,43,44].

The physician is independent in choosing the therapy, even by being able to deviate from the guidelines if they are not applicable to the specific case, as reaffirmed in Italy by the Gelli–Bianco law. Therefore, the first element to be assessed is whether the proposed therapy is relevant to the needs of the case. After checking the appropriateness of the therapy, it is necessary to assess the safest treatment options for the patient among the available treatment options. This tool did not reduce the development of VTE or adverse events but made women much more aware of the choice made and the risks associated with COC treatment. In our experience, approximately 89% of patients were satisfied with using informed consent before prescribing COC treatment as they felt more medically protected. On the other hand, in all cases, the patients expressed an opinion in favor of signing an informed consent, thus feeling involved and more aware of the choice undertaken. The conduct of a physician who prescribes, albeit diligently, therapy that is riskier for the patient, if it results in damage, is considered negligent as they have discarded other therapeutic options suitable for the specific clinical condition and such as to avoid the determination of the harmful event (sentence n. 8875 of 08.09.1998 of the Cassation Criminal Section III). The treatment provider should actively investigate the patient’s risk factors. Therefore, diagnostic investigations are useful in case of doubt. Communication with the patient must address both the personalized risk and the benefits of the therapy. This communication must be in writing and sufficiently clear as well as appropriate to the patient’s cultural level.

## 5. Conclusions

Contraception is a delicate, sometimes difficult issue which carries many ethical, moral, and medical dilemmas. Contraceptive counselling should begin early, and the choice of method based on the impact of (an unplanned) pregnancy, the risks, and benefits of the contraceptive type and the individual’s preferences. Therefore, generic, or overly technical consent forms are invalid, so they cannot be understood by the patient. Therefore, while some generic information may be written on a pre-filled consent form, there is a need to allow for individualization of information on a case-by-case basis, ensuring that the written consent form is clear in all its essential parts: (i) therapeutic purposes, (ii) therapeutic alternatives and their risk profiles, (iii) general and specific side effects. Through this study, we have highlighted how informed consent used before the onset of COS therapy is useful to the doctor from the point of view of forensic protection (85% of doctors readily agreed), but in all the patients, it has been evaluated as a useful tool for being properly informed. This allows women to recognize the side effects of therapy early but more importantly to initiate the treatment choice more consciously. Behavioral measures must be taken to avoid treatment failure and complications.

## Data Availability

Data is contained within the article and supplementary material.

## Supplementary Materials

The following are available online at https://www.mdpi.com/article/10.3390/jpm13040584/s1, Proposal of informed consent targeted to COC prescription.
