# Supplementary material for: A Multi-Institutional Informed Consent Proposal as a Prevention Tool for Combined Oral Contraceptive Intake and Thrombotic Risk
Source: J Pers Med. 2023 Mar 27;13(4):584. doi: 10.3390/jpm13040584 (PMC10144716; doi:10.3390/jpm13040584)
Supplement: Supplementary file 1 [file jpm-13-00584-s001.zip › jpm-2174111-supplementary.pdf]

INFORMED CONSENT FORM ON COC (Combined Oral Contraceptive)

HOW TO TAKE IT

Taking combined hormonal contraception (COC), from 1 day of Menstrual Cycle ensures immediate effectiveness. Wanting to start taking at any time of the cycle, it is recommended to use an additional contraception for 7 days and, only with the pill with Estradiol Valerate (EV) + Dienogest (DNG) in the formulation 26+2, for 9 days.

WARNINGS

The first week of taking contraception, either combined estrogen-progestin oral contraceptive pills (OCPs) or progestogen-only pills (POPs), inhibits ovulation. Delays or forgetting to take pills considerably increases the chances of pregnancy during the first week of the pack. The allowed intake delay is 12 hours, after 24 hours it is advisable to take two pills together. Risk of ineffectiveness in case of vomiting within 3 hours or profuse diarrhea after 4-6 hours of intake. First clinical check-up after 12 months from the beginning of therapy.

Control of blood pressure within 6 months of starting therapy. COC use should be discontinued 4 weeks before major surgery.

SIDE EFFECTS

COC are associate with an increase risk of venous thromboembolism (VTE) but it should be considered that, in most women, the benefits associated with using COC far outweigh the risk of serious side effects. Possibility of water retention that can cause an increase in weight of not more than 2kg, totally reversible to the suspension of the method. However, the available literature is not sufficient to determine the effect of COC on weight. Sensation of heavy legs, headache while taking or during the break period, mastodynia, nausea, rarely vomiting, changes in sexual desire. Most side effects are reduced already from the second cycle, often resolving spontaneously within 5 months. Occasional bleeding may occur. Have specialized medical assessment in the case of side effects of any entity.

Please note: COC exposes to an increased risk of venous and arterial thrombosis ranging from 5 to 12 per 10,000 women each year depending on the type of COC taken, therefore it is recommended to alert the doctor and to go to the emergency department if there are signs or symptoms attributable to this event (swollen arms or ankles, legs tingling, lower extremity edema, erythromelalgia, dyspnea, etc). In this regard, it is recommended a periodic re-evaluation of individual risk factors such as age, smoking, obesity, etc.

SYMPTOMS OR DIAGNOSES REQUIRING CLINICAL ASSESSMENT DURING COC.

- Urgent action: chest pain, shortness of breath, hemoptysis, pain in one leg (usually the calf or inner thigh), swelling in the leg or arm, numbness or weakness on one side of the body, sudden change in your mental state.
- Outpatient care: breast node, secretion from the nipple, nipple inversion, changes in the breast skin; new onset migraine, new sensory or motor symptoms in the hour preceding the onset of migraine; persistent atypical vaginal bleeding; arterial hypertension, increase in body mass index (>35 kg/m2) (BMI >35), migraine or migraine with aura, deep venous thrombosis or pulmonary embolism, blood clotting abnormality, antiphospholipid antibodies positivity;, angina, heart attack, stroke or peripheral vascular disease, atrial fibrillation, cardiomyopathy; mutation of the gene BRCA1-2 or breast cancer, liver tumor, gallbladder calculus.

EXTRA-CONTRACEPTIVE BENEFITS

Hormonal contraception exerts a protective effect on genital integrity and fertility achieving overall a benefit in terms of reproductive health. Reduction of dysmenorrhea and heavy menstrual bleeding, premenstrual syndrome, menstrual headache, hyperandrogenic symptoms, endometriosis, ectopic pregnancy, pelvic inflammatory disease (PID), prevention of ovarian, endometrial and colorectal cancers.

Date________________

Personal medical records of

____

(name, family name, date and place of birth)

Weight________________________Heigh_______________________________ Blood pressure____________________

Exclusion for medical history of____

____

**<u>Absolute</u> contraindication to the use of COC**	YES	NO
Systolic arterial hypertension ≥ 160 mmhg and diastolic ≥ 100 mmhg or with presence of organ damage.		
Ischemic or dilated cardiomyopathy, complicated valvulopathy, in current or past.		
Current or past stroke.		
Current or previous venous thromboembolism/pulmonary embolism or on anticoagulant therapy.		
Thrombophilic gene mutations.		
Diabetes for more than 20 years or with organ failure.		
Cigarette smoke in women >35 yo and >15 cigarettes a day.		
Multiple cardiovascular risk factors.		
Migraine with aura.		
Migraine without aura in women > 35years.		
Major surgery with prolonged immobilization.		
Present or previous malignant breast cancer <5 years.		
Malignant liver neoplasms.		
Acute viral hepatitis.		
Liver decompensated cirrhosis.		
Complicated organ transplantation.		
Systemic Lupus Erythematosus with positive or unknown antiphospholipids antibodies.		
Lactation <6 weeks after delivery.		
Post-partum <21 days after delivery.		
Endometrial cancer.		
First-degree relatives, as well as brothers or sisters with risk factors (cardiovascular diseases, in particular thromboembolism, known thrombophilia, adverse obstetric events, diabetes, hyper/dyslipidemia, connectivitis, autoimmune diseases) insurgents before age 50.		

The patient

____

(name, family name, date and place of birth)

has been provided through information by the Doctor of Medicine (MD) practitioner

____

(name and family name)

as to the advantages and risks of COC intake and any contraindications has been evaluated before prescription.

Date and place

Signature of MD practitioner who provided the information

Signature of the patient
